# One-year outcome of combination therapy with intravitreal aflibercept and photodynamic therapy for polypoidal choroidal vasculopathy

**DOI:** 10.1186/s40360-019-0310-1

**Published:** 2019-05-14

**Authors:** Hsin-Yu Weng, Tzu-Lun Huang, Pei-Yao Chang, Jia-Kang Wang

**Affiliations:** 1 Department of Ophthalmology, Far Eastern Memorial Hospital, New Taipei City, Taiwan; 2 Department of electrical engineering, Yuan Ze University, Taoyuan City, Taiwan; 3 Department of Medicine, National Taiwan University, Taipei City, Taiwan; 4 Department of Medicine, National Yang Ming University, Taipei City, Taiwan; 5 Department of Healthcare Administration and Department of Nursing, Oriental Institute of Technology, New Taipei City, Taiwan

**Keywords:** Intravitreal injection, Aflibercept, Photodynamic therapy, Polypoidal choroidal vasculopathy

## Abstract

**Background:**

To investigate the one-year visual and anatomical outcomes of combination therapy with intravitreal aflibercept (IVA) and photodynamic therapy (PDT) for treating polypoidal choroidal vasculopathy (PCV).

**Methods:**

This was a retrospective case-series study, including 30 eyes from 30 patients with treatment-naïve PCV treated by combination therapy with IVA and PDT. Best-corrected visual acuity (BCVA), central retinal thickness (CRT), complete polyp regression rate, and dry macula rate were recorded every 3 months during 12-month follow-up. Clinical factors associated with final visual outcome and retreatment were investigated.

**Results:**

The mean LogMAR BCVA was significantly improved from 0.73 ± 0.65 at baseline to 0.51 ± 0.60 (*p* = 0.01), and the mean CRT was also significantly improved from 339 ± 96 μm at baseline to 244 ± 43 μm at 12-month follow-up (*p* <  0.001). Complete regression of polypoidal lesions was 76.7%, and dry macula rate was 100% at 12 months. Better final BCVA was associated with younger age and better baseline BCVA (*p* = 0.02 and p <  0 001). The patients without complete polyp regression at 3-month follow-up were associated with retreatment (*p* = 0.03).

**Conclusion:**

In this study, combination therapy with IVA and PDT had significant visual and anatomical improvements to PCV patients during one-year follow-up. Better baseline BCVA and younger age were found to be associated with better visual outcome.

**Electronic supplementary material:**

The online version of this article (10.1186/s40360-019-0310-1) contains supplementary material, which is available to authorized users.

## Summary

Combination therapy with intravitreal aflibercept and photodynamic therapy had significant visual and anatomical improvements for patients with polypoidal choroidal vasculopathy during one-year follow-up. Better baseline visual acuity and younger age were found to be associated with better visual outcome.

## Background

Polypoidal choroidal vasculopathy (PCV) is a subtype of exudative age-related macular degeneration (AMD), and is characterized by polyp-like aneurismal nodules with or without branching vascular networks (BVN) on indocyanine-green angiography (ICGA) [1]. The prevalence of PCV is higher in Asians than in Caucasians [2]; therefore, the disease is important in Asian populations, including in Taiwan.

The natural course and visual prognosis of PCV is better than other subtypes of exudadtive AMD [3]. However, some patients may experience marked bleeding and leakage and worse visual outcome [4]. Current treatment modalities for PCV include photodynamic therapy (PDT) and intravitreal injection of anti-vascular endothelial growth factor (anti-VEGF) agents. PDT has favorable visual outcome and regression of the polyps in treating PCV [5]; however, it causes hypoxia in retinal pigment epithelium, leading to enhanced VEGF and angiogenesis [6]. Anti-VEGF is beneficial in vision outcome, but the polyp regression rate is relative low for PCV [7].

Combination of PDT and anti-VEGF is considered to have synergistic effects of visual improvement and polyp regression. The EVEREST study reported PDT combined with intravitreal ranibizumab (IVR) or PDT alone had superior effect on polyp regression than IVR monotehrapy during 6-month follow-up [8]. Additionally, a systematic review and meta-analysis reported that PDT combined with anti-VEGT had better visual acuity and lower retinal hemorrhage than PDT alone [9].

Aflibercept is a recombinant fusion protein which binds to VEGF-A, VEGF-B and placental growth factor, and is more potent and prolonged effect than ranibizumab or bevacizumab [10]. Previous study reported IVA had comparable visual outcome and more polyp regression compared with IVR [11]. Additionally, IVA is effective for patients with PCV refractory to IVR [12].

This study was conducted to evaluate the one-year visual and anatomical outcomes of combination therapy with IVA and PDT for PCV. In addition, baseline characteristics that could be associated with better visual improvement were identified.

## Methods

We retrospectively reviewed the medical records of 30 eyes from 30 patients with symptomatic subfoveal PCV that were treated with combination therapy of IVA and PDT at Far Eastern Memorial Hospital in Taiwan between August 2015 and December 2017. This study was approved by the Institutional Review Board at Far Eastern Memorial Hospital (ethical approval office reference number: FEMH-107160-E), and was conducted in accordance with the Declaration of Helsinki. Written informed consent was obtained from each patient prior to treatment.

The diagnosis of subfoveal PCV was made by polypoidal choroidal vascular lesion with or without a BVN at subfoveal area on ICGA. All patients were treatment-naïve and were followed for 12 months. The exclusion criteria were patients with diabetic retinopathy, retinal arterial or venous occlusion, retinal detachment, and intraocular inflammation or infection.

Before starting treatments, all patients underwent comprehensive ophthalmic examinations, including Best-corrected visual acuity (BCVA) using Snellen E chart, slit-lamp biomicroscopy and fundus exam, central retinal thickness (CRT) by optical coherence tomography (OCT) (RTVue; Optovue Inc., Fremont, California, USA), fluorescein angiography (FA), and ICGA (HRA; Heidelberg Engineering, Heidelberg, Germany).

All subjects received intravitreal injection of 2.0 mg aflibercept in 0.05 mL followed by standard PDT within a week. The standard PDT was administered according to Treatment of Age-Related Macular Degeneration with Photodynamic Therapy studies [13]. After the combination treatment, patients received examinations including BCVA, slit-lamp biomicroscopy, and OCT at 3, 6, 9, and 12 months follow-up. FA and ICGA were repeated 3 months after the initial treatment, and 3 months after additional combination therapy.

Retreatment included combination therapy and IVA monotherapy. Combination therapy with IVA and PDT was performed if exudative changes such as subretinal or intraretinal fluid were found by OCT, and residual or recurrent polypodial vascular lesions were found by ICGA. If there were exudative changes without residual or recurrent polyp, IVA monotherapy was performed repeatedly until the macula becoming dry. The number and timing of retreatment were recorded. Retreatment rate was defined as patients receiving either second combination therapy or IVA monotherapy of all participants.

Outcomes measures included the changes in BCVA, the changes in CRT, the rate of complete polyp regression, and the rate of dry macula. The visual acuity was converted from a Snellen E chart to a logarithm of the minimum angle of resolution (LogMAR) value for statistical analysis. Patients with visual gain or visual loss ≥0.3 LogMAR units were recorded. Dry macula means no intraretinal or subretinal fluid at macula on OCT.

All statistical analyses were performed using SPSS (V. 20.0; IBM, Armonk, New York, USA). The paired t-test was used to determine the significance of the difference of LogMAR BCVA and CRT between baseline and 3, 6, 9, 12 months follow-up. The correlations of clinical factors and visual outcome were tested by Pearson’s correlation coefficient. *P*-values less than 0.05 were considered statistically significant.

## Results

Table 1 presents baseline characteristic data of 30 eyes of the 30 patients included in this study. The mean age was 65.5 ± 12.8 years (range: 40–92 years). There were 23 male patients (76.7%) and 7 female patients (23.3%).

**Table 1:** Baseline characteristics of the participants

Eye (right eye %)	18 (60%)
Gender (male %)	23 (76.7%)
Age, Mean (±SD) (years)	65.5 (12.8)
Baseline LogMAR BCVA (±SD)	0.73 (0.65)
Baseline CRT (±SD) (μm)	339 (96)
Multiple polyps (≧2)	5 (16.7%)
Polyp location (subfovea)	19 (63.3%)

*SD* standard deviation

*LogMAR* logarithm of the minimum angle of resolution,

*BCVA* best corrected visual acuity

*CRT* central retinal thickness

**Fig. 1:**
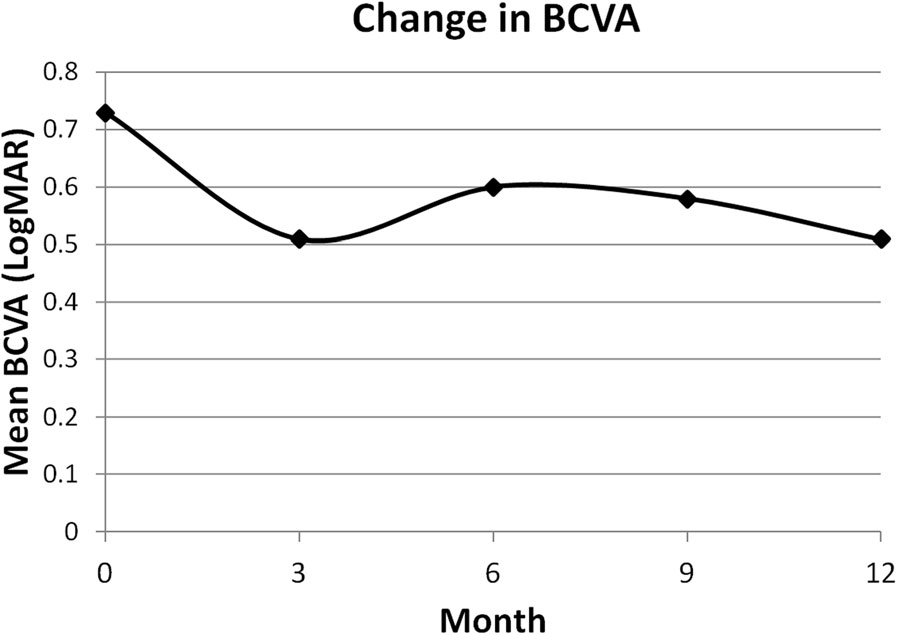
Change in mean Best-corrected visual acuity (BCVA) in LogMAR

The mean logMAR BCVA significantly improved from 0.73 ± 0.65 at baseline to 0.51 ± 0.50 at 3 months (*p* = 0.002), to 0.60 ± 0.69 at 6 months (*p* = 0.045), to 0.58 ± 0.65 at 9 months (p = 0.045), and to 0.51 ± 0.60 (*p* = 0.01) at 12 months (Fig. 1).

At 3 months of follow-up, 8 eyes (26.7%) had an improvement in BCVA of ≥0.3 LogMAR units, and no eye had a decrease in BCVA of ≥0.3 LogMAR units. At 12 months of follow-up, 10 eyes (33.3%) had an improvement in BCVA of ≥0.3 LogMAR units, and 2 eyes (6.7%) had a decrease in BCVA of ≥0.3 LogMAR units. The number of eyes with a visual acuity of 20/40 or better increased from 9 eyes (30%) at baseline to 13 eyes (43.3%) at 3 months and to 16 eyes (53.3%) at 12 months (Table 2).

**Table 2:** Changes in visual and anatomical outcomes

	Baseline	3 months	*p* value	12 months	p value
Mean BCVA (LogMAR)	0.73	0.51	0.002	0.51	0.01
Mean CRT (μm)	339	249	< 0.001	244	< 0.001
Complete regression of polyps		16 (53.3%)		23 (76.7%)	
Dry macula		28 (93.3%)		30 (100%)	
BCVA improvement (≥ 0.3 LogMAR units)		8 (26.7%)		10 (33.3%)	
BCVA loss (≥ 0.3 LogMAR units)		0 (0%)		2 (6.7%)	

*LogMAR* logarithm of the minimum angle of resolution

*BCVA* best corrected visual acuity

*CRT* central retinal thickness

*P* value: comparing to baseline

**Fig. 2:**
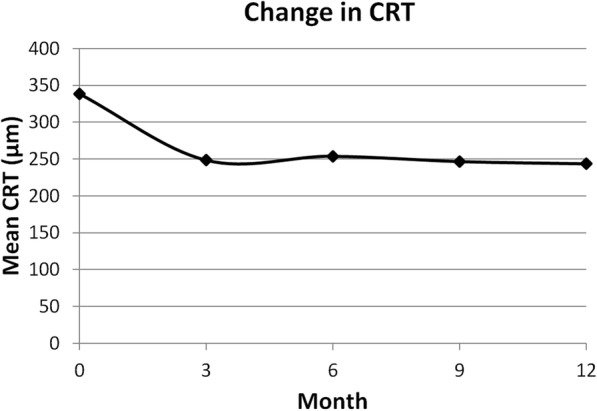
Change in mean central retinal thickness (CRT)

The mean CRT decreased significantly from 339 ± 96 μm to 249 ± 38 μm at 3 months, to 254 ± 50 μm at 6 months, to 247 ± 47 μm at 9 months, and to 244 ± 43 μm at 12 months (*p* <  0.001 at all time-points follow-up) (Fig. 2). At 3 months after the treatment, 16/30 eyes (53.3%) showed complete regression of polypoidal lesions on ICGA, while 14/30 eyes (46.7%) showed partial regression. At 12-month follow-up, there were 23/30 eyes (76.7%) with complete regression, and 7/30 eyes (23.3%) with partial regression. Dry macula was achieved in 28 eyes (93.3%) and 30 eyes (100.0%) at 3 and 12 months after treatment, respectively (Table 2).

Final BCVA was associated with age (*r* = 0.425, *p* = 0.02) and baseline BCVA (*r* = 0.750, *p* <  0.001). There was no association of final BCVA with gender, polyp number, polyp distribution (solitary or clustered), polyp location (subfoveal, juxtafoveal, or extrafoveal), baseline CRT, dry macula, or complete regression of polyps. Change of BCVA during the 12-month follow-up was only associated with baseline BCVA (*r* = 0.455, *p* = 0.01).

The mean treatment number was 3.1 courses, which included 1.6 courses of IVA and 1.5 courses of PDT. Retreatment was performed in 50% (15/30) of patients. Combined retreatments were all aimed for eyes with residual non-regressed polyps, not newly developed choroidal vascular polyps. There were no significant differences of final BCVA and final CRT between eyes with retreatment and eyes without treatment (*p* = 0.23 and 0.85, respectively). The patients without complete polyp regression at 3-month follow-up were associated with retreatment (*p* = 0.03). There was no correlation between retreatment and other baseline factors.

Table 3 presents associations between clinical factors and the visual outcome. No ocular or systemic adverse events were detected or self-reported during the follow-up period in this study. The raw data were attached as Additional file 1.

**Table 3:** Associations between clinical factors and the visual outcome

Factors	Association (r value)	Significance (p value)
Age	0.425	0.02
Baseline LogMAR BCVA	0.750	< 0.001
Baseline CRT	0.153	0.42
Polyp number	0.303	0.10
Polyp distribution	0.121	0.53
Polyp location	0.039	0.84

*LogMAR* logarithm of the minimum angle of resolution

*BCVA* best corrected visual acuity

*CRT* central retinal thickness

## Discussion

To our knowledge, this is the first report of one-year outcome of combination therapy with IVA and PDT in PCV which only included patients in Taiwan. The combination therapy showed favorable effects including the visual improvement and polyp regression of PCV cases.

Previous studies have reported remarkable visual and anatomical improvement of combination therapy with IVA and PDT for treating PCV patient. Matsumiya et al. [14] reported 0.10 and 0.12 LogMAR BCVA gain at 3 months and 12 months follow-up, respectively. Kikushima et al. [15] reported 0.25 and 0.30 LogMAR BCVA gain at 3 months and 12 months of follow-up, respectively. In the present study, the visual improvement was comparable to prior studies. After combination therapy, the mean LogMAR BCVA improved by 0.22 (from 0.73 to 0.51, *p* < 0.001) at both 3-month and 12-month follow-up.

As for dry macula, Matsumiya et al. [14] reported 70 and 90% of dry macula rate at 3 months and 12 months follow-up, respectively. In this present study, the dry macula rates were 93.3 and 100% at 3 months and 12 months of follow-up, respectively, which were better than that reported previously. The improvement in the mean CRT was also comparable to the values in previous studies for IVA monotherapy or combination therapy of anti-VEGF and PDT.

After IVA + PDT, Matsumiya et al. [14] reported 70 and 78% of complete regression rate at 3 months and 12 months follow-up, respectively, and Kikushima et al. [15] reported 87.5 and 68.8% of complete regression rate at 3 months and 12 months follow-up, respectively. In this study, combination therapy with IVA and PDT achieved complete polyp regression in 53.5% of PCV cases at 3 months of follow-up, and in 76.7% of cases at 12 months of follow-up. The 3-month polyp regression rate was inferior to that of previous studies, but the 12-month polyp regression rate was comparable between to that of prior studies.

Matsumiya et al. [14] reported 90% of dry macula rate and 78% of complete polyp regression rate at 12-month follow-up after combined IVA and PDT treatment. In this study, similar anatomical outcomes were observed as 100% of dry macular rate and 76.7% of complete polyp regression rate at Month 12 following the same combined therapy. The above facts indicated that there were residual choroidal vascular polyps without active fluid leakage after combined treatment. It is mandatory for these patients with incompletely regressed polyps to closely follow-up of recurrence of accumulation of subretinal or/and intraretinal fluid.

The anatomical outcomes including dry macula and complete regression of polypoidal lesion may be related with the treatment number. In previous study, the mean number of injection through 12 months was 3.9 ± 1.9 in IVA monotherapy for PCV patients [12]. In another study of IVA + PDT, the mean number of treatment was 3.7 courses, which included 2.2 IVA + 1.5 PDT [14]. In the present study, the mean treatment number was 3.1 courses, which included 1.6 courses of IVA and 1.5 courses of PDT. The number of IVA in this study was less than that in the previous study.

We investigated the clinical factors associated with the final visual outcome. Younger had better visual outcome in this study. A recent 2-year study reported that absence of retreatment was associated with younger age and female [18]. Better baseline BCVA was also associated with good visual outcome in our study, and the same result was also reported by other studies [15–17]. Moreover, baseline smaller greatest linear dimension was found as a significant predictor for good visual improvement in previous reports [14, 15, 17]. Besides, greater subfoveal choroidal thickness was associated with better BCVA, and lesser subfoveal choroidal thickness was associated with retreatment in a previous report [17].

There were several limitations in this study. First, the present study was retrospective in nature and there were confounding factors, and it was a single arm design. Second, our case number was relatively small. Third, not all patients were treatment-naïve.

## Conclusion

Combination therapy with IVA and PDT had significant visual and anatomical improvements for PCV patients during one-year follow-up. Better baseline BCVA and younger age were found to be associated with better visual outcome.

## Additional file

Additional file 1: The raw data of the study**.** “One-year outcome of combination therapy with intravitreal aflibercept and photodynamic therapy for polypoidal choroidal vasculopathy“ (XLSX 32 kb)

## Abbreviations

BCVA: Best-corrected visual acuity
CRT: Central retinal thickness
IVA: Intravitreal aflibercept
PCV: Polypoidal choroidal vasculopathy
PDT: Verteporfin photodynamic therapy
